# Supplementary material for: Hyperglycemia aggravates microenvironment hypoxia and promotes the metastatic ability of pancreatic cancer
Source: Comput Struct Biotechnol J. 2018 Oct 29;16:479–87. doi: 10.1016/j.csbj.2018.10.006 (PMC6232646; doi:10.1016/j.csbj.2018.10.006)
Supplement: Supplementary file 1 — Supplementary material [file mmc1.doc]

**Hyperglycemia aggravates microenvironment hypoxia and promotes the metastatic ability of pancreatic cancer**

Wei Li^1^, Han Liu^2^, Weikun Qian^1^, Liang Cheng^1^, Bin Yan^1^, Liang Han^1^, Qinhong Xu^1^, Qingyong Ma^1^*, Jiguang Ma^3^*

^1^Department of Hepatobiliary Surgery, First Affiliated Hospital of Xi’an Jiaotong University, Xi’an, 710061, China

^2^ Department of Hepatobiliary Surgery, Qilu Hospital of Shandong University, Shandong, 250012, China

^3^Department of Anesthesiology, First Affiliated Hospital of Medical College, Xi’an Jiaotong University, Xi’an, 710061, China

* Corresponding authors. Address: Department of Anesthesiology (J. Ma) or Department of Hepatobiliary Surgery (Q. Ma), First Affiliated Hospital, Xi’an Jiaotong University, 277 West Yanta Road, Xi’an Shaanxi 710061, China.

Tel: +86 29 8532 3899; Fax: +86 29 8532 3899

E-Mail: [jgma86@mail.xjtu.edu.cn](mailto:jgma86@qq.com)  (J. Ma) or [qyma56@mail.xjtu.edu.cn](mailto:qyma56@mail.xjtu.edu.cn) (Q. Ma)

**Abstract**

**Background:** Diabetes mellitus and pancreatic cancer are intimately related. Our previous studies showed that high levels of blood glucose promote epithelial-mesenchymal transition of pancreatic cancer. In this study, we evaluated the relationship between hyperglycemia and hypoxic tumor microenvironments.

**Methods:** HIF-1α expression was evaluated by immunohistochemistry in clinical pancreatic cancer tissues with or without diabetes mellitus. Statistcal analysis was performed to explore the relationship between HIF-1α expression and pathological features of patients with pancreatic cancer. *In vivo* and *in vitro* models was established to detect whether a hyperglycemia environment could cause hypoxia in the pancreatic parenchyma and promotes pancreatic cancer. In addition, we also tested the effect of HIF-1α siRNA on the high glucose-induced invasive and migratory abilities of BxPC-3 cells in culture.

**Result:** Our data showed that pancreatic cancer patients with diabetes had a higher level of HIF-1α expression as well as biliary duct invasion and larger tumor volumes than individuals in the euglycemic group. Diabetic nude mice treated with streptozotocin (STZ) exhibited larger tumors and were more likely to develop liver metastasis than control mice. Acinar cells of the pancreas in diabetic mice showed an obvious expansion of the endoplasmic reticulum and increased nuclear gaps as well as chromatin close to the cellular membrane in some acinar cells. The expression area for Hypoxyprobe-1 and HIF-1α in the diabetic orthotopic xenograft group was larger than that in the control group. The expression level of HIF-1α in the BxPC-3 cancer cell line increased in response to high glucose and CoCl_2_ concentrations. The high glucose-induced invasive ability, migratory capacity and MMP-9 expression were counter-balanced by siRNA speciﬁc to HIF-1α.

**Conclusion:** Our results demonstrate that the association between hyperglycemia and poor prognosis can be attributed to microenvironment hypoxia in pancreatic cancer.

**Key words:** Hyperglycemia, hypoxia, HIF-1α, metastasis, pancreatic cancer

**Background**

Pancreatic cancer is one of the deadliest types of malignant carcinoma and is typically diagnosed at a late stage, with a 3% 5-year survival rate in the US [1]. In China, pancreatic cancer is the sixth leading cause of cancer death, and the estimated numbers of newly diagnosed cases and deaths were 80,344 and 72,723, respectively, in 2011 [2]. Surgical resection remains the best chance at long-term survival for pancreatic cancer. However, most patients are diagnosed with the unresectable form because of metastasis [3]. The causes of pancreatic cancer are still not known, although certain risk factors have been identified, including smoking, obesity, and diabetes mellitus [4].

Evidence is accumulating that diabetes is associated with pancreatic cancer development and progression as well as death in pancreatic cancer patients [5-6]. However, the mechanism of this phenomenon remains unknown. Our previous studies found that high glucose levels could promote pancreatic cancer proliferation and invasion as well as epithelial-mesenchymal transition (EMT) and metastasis [7-8]. Recent studies have proven that hyperglycemia can induce cellular hypoxia and generate mitochondrial reactive oxygen species [9].

 Pancreatic tumors are generally hypoxic due to their avascular morphology [10]. In a hypoxic environment, pancreatic cancer cells express high levels of the hypoxia-inducible factor 1α (HIF-1α). The target genes of HIF-1α encourage an aggressive phenotype, promoting tumor growth, invasion and metastasis [11]. Hyperglycemia and cellular hypoxia are intimately related. In tumor cells, high glucose levels are known to promote HIF-1α expression under both normoxic and hypoxic conditions [12]. In this study, we assumed that hyperglycemia promotes the progress of pancreatic cancer by inducing a hypoxic microenvironment by regulating HIF-1α.

To test this hypothesis, the expression of HIF-1α and the clinical and pathological features of patients with pancreatic cancer in the First Hospital of Xi’an Jiaotong University were analyzed. The diabetes orthotopic xenograft model was also established to detect whether a hyperglycemia environment could cause hypoxia in the pancreatic parenchyma and lead to pancreatic cancer. In addition, we also tested the effect of HIF-1α siRNA on the high glucose-induced invasive and migratory abilities of BxPC-3 cells in culture. By investigating the underlying mechanism, we hope to find evidence to explain how diabetes facilitates pancreatic cancer progression.

**Methods**

*Collection of tissues*

From January 2012 to December 2014, 105 pancreatic ductal carcinomas were subjected to clinical examination at the First Affiliated Hospital of Xi’an Jiaotong University, China. Most of the patients were diagnosed at an advanced stage and were not suitable for surgical resection. Only 46 patients, who received a radical curative pancreatic operation with a pathologic diagnosis, and 8 normal pancreases were included in this study. The pancreatic specimens were divided into two groups according to the fasting blood glucose levels of the patients (an average long-term glucose level for patients with a history of diabetes mellitus and an average three-day glucose level after hospitalization): (1) euglycemia and (2) hyperglycemia. The study protocol and consent forms conform to the Declaration of Helsinki and were approved by the Ethical Review Board (ERB) Committee of The First Affiliated Hospital of Xi’an Jiaotong University, China. Written informed consent was obtained from all the participants.

*Cell Culture and Reagents*

The BxPC-3 cell line, obtained from the American Type Culture Collection (Manassas, VA, USA), was cultured in Dulbecco’s Modified Eagle’s Medium (DMEM) containing 10% dialyzed, heat inactivated, fetal bovine serum (FBS) and 1% penicillin-streptomycin (Sigma Aldrich, St Louis, MO, USA) in a 95% air/5% CO_2_ humidified atmosphere at 37˚C. Both DMEM and FBS were purchased from HyClone (Logan, UT, USA). Streptozotocin (STZ) was acquired from Sigma Aldrich (St. Louis, MO, USA). The primary antibodies against HIF-1α and MMP-9 were procured from Santa Cruz Biotechnology (Santa Cruz, CA, USA). Millicell Transwells for the invasion assays were obtained from Millipore (Billerica, MA, USA). Matrigel was from BD Biosciences (Bedford, MA, USA). Other reagents were purchased from common commercial sources. All drug solutions were freshly prepared on the day of testing.

*Diabetes Mouse Model and Orthotopic Tumor Model*

The 5-week-old BALB/c athymic nude mice (male) were purchased from the Shanghai Experimental Animal Center. Animal care and experiments were carried out in accordance with the guidelines of the Xi’an Jiaotong University. The nude mice were grouped into euglycemic and hyperglycemic groups (n = 10). The hyperglycemic mice each received an intraperitoneal injection of STZ (dissolved in sodium citrate buffer) at a dose of 175 mg/kg body weight. Blood glucose levels were determined with an ACCU-CHEK Active meter. BxPC-3 cells (1 × 10^8^) were injected in a total volume of 50 μl PBS into the body of the pancreas. The mice were sacrificed after 8 weeks, and the tumors, livers and pancreases were collected and analyzed. All experimental protocols were approved by the Ethical Committee of the First Afﬁliated Hospital of Xi’an Jiaotong University, Xi’an, China.

*Immunohistochemistry*

Formalin fixed and paraffin embedded pancreas and pancreatic cancer tissue samples were used for the immunohistochemistry tests. In brief, the tissue sections were incubated with primary antibodies (HIF-1α, 1:50; pimonidazole, hypoxyprobe-1 Mab, 1:100) overnight at 4°C and then incubated with the appropriate biotinylated secondary antibody. The results were visualized using DAB, and the slides were counterstained with hematoxylin. The densitometry analysis of the immunohistochemical staining was performed using the Image-Pro Plus 6.0 software.

*Transmission Electron Microscopy (TEM)*

For transmission electron microscopy analyses, pancreas samples were initially fixed in 2.5% glutaraldehyde in PBS at 4°C overnight, post-fixed in 1.0% osmium tetroxide for 2 h, dehydrated and embedded in Epon 812 resin, and then cut into semithin sections (1-2 μm). Following methylene blue staining, the semithin sections were cut into thin sections (70 nm) using an ultrathin microtome. Finally, thin sections were lightly counterstained with uranyl acetate and lead citrate and examined with TEM (H-7650, Hitachi, Tokyo, Japan).

*Transwell Matrigel invasion assay*

The 8.0 μm pore inserts were coated with 30 μl of Matrigel. BxPC-3 cell suspensions (5×10^4^) were added to the upper chambers in DMEM containing 1% FBS. DMEM containing 20% FBS was placed in the lower chambers. After being incubated for 48 h, the non-invading cells were removed by scraping with a wet cotton swab, and the invading cells were stained with crystal violet. The invasion ability was determined by counting the stained cells on the bottom surface.

*Wound healing assay*

BxPC-3 cells were seeded in 24-well plates (1.0 × 10^5^ cells/500 µl). A sterile pipette tip was used to produce a wound line between cells after the cells grew to 90-100% confluence. Then, the cells were allowed to migrate for 24 h. Images were taken at time 0 and 24 h post-wounding using a Nikon Diaphot TMD inverted microscope (×10).

*RNAi transfections*

siRNA against HIF-1α (5’- CCA CCA CUG AUG AAU UAA ATT -3’, 5’- UUU AAU UCA UCA GUG GUG GTT’) and a negative control siRNA (NC: 5’- UUC UCC GAA CGU GUC ACG UTT -3’, 5’- ACG UGA CAG GUU CGG AGA ATT -3’) were purchased from GenePharm (Shanghai, China).

*Real-time quantitative PCR (qRT-PCR)*

Total RNA extracted from BxPC-3 cells (Fastgen200 RNA isolation system, Fastgen, Shanghai, China) was reverse-transcribed into cDNA using the PrimeScript RT reagent Kits (TaKaRa, Dalian, China). The primer sequences were as follows:

MMP-9-F: 5’- GCA ATG CTG ATG GGA AAC CC -3’.

MMP-9-R: 5’ AGA AGC CGA AGA GCT TGT CC -3’.

β-actin-F: 5’-GAC TTA GTT GCG TTA CAC CCT TTC T -3’.

β-actin-R: 5’- GAA CGG TGA AGG TGA CAG CAG T -3’.

The PCR reactions consisted of 30 s at 95°C, followed by 40 cycles of 95°C for 5 s, 60°C for 30 s and 72°C for 30 s. The relative gene expression was calculated using the previously described 2^-ΔΔCt^ method [13].

*Western blot analysis*

Proteins were electrophoretically resolved on a denaturing SDS-polyacrylamide gel and were electrotransferred onto polyvinylidene difluoride membranes. The membranes were blocked with 5% nonfat dry milk in Tris-buffered saline (TBS) for 2 h and then probed with HIF-1α and MMP-9 antibodies at 4°C overnight. After being blotted with the secondary antibody for 2 h at 37°C, the complexes were visualized using the ECL Western blotting substrate and photographed by ChemiDoc XRS imaging system (Bio-Rad, USA).

*Statistical analysis*

Statistical analysis was performed using SPSS software (version 17.0, SPSS Inc., Chicago, USA). Data are presented as the means ± SD of three replicate assays. Differences between the groups were analyzed by the Chi-square test, Student's *t*-test or analysis of variance (ANOVA). Statistical significance was set at P < 0.05. All experiments were repeated independently at least three times.

**Results**

***Diabetes promotes the progression of pancreatic cancer in patients and HIF-1α protein synthesis.***

A total of 105 pancreatic cancer patients were collected in the First Hospital of Xi’an Jiaotong University. Among these, 46 patients (43.8%) were treated with surgical resection and had complete pathological information. Patients were divided into hyperglycemic and euglycemic groups. The tumor size, invasion and lymph node metastasis of these patients are listed in **Table 1**. Of these 46 patients, 18 (39.1%) were diagnosed with diabetes, and 28 (60.9%) had normal glucose levels. We observed that patients in the hyperglycemic group had slightly higher levels of peripancreatic fat tissue invasion (66.7% vs 57.1%), vascular invasion (50% vs 42.9%) and lymph node metastasis (27.8% vs 14.3%) than those in the euglycemic group. We also found that pancreatic cancer patients with diabetes had significantly more biliary duct invasion and greater tumor volumes than patients in the normal blood glucose group. Our clinical data indicated that pancreatic cancer was more invasive in hyperglycemic patients than in euglycemic patients.

**Table 1. Tumor size and invasion of pancreatic cancer patients with or without diabetes.**

|  | Hyperglycemia（18） | Euglycemia（28） | *P* |
| --- | --- | --- | --- |
| Peripancreatic fat tissue invasion | 12 (66.7%) | 16 (57.1%) | 0.518 |
| Lymph node metastasis | 5(27.8%) | 4 (14.3%) | 0.284 |
| Vascular invasion | 9 (50%) | 12 (42.9%) | 0.635 |
| Biliary ducts invasion | 4 (22.2%) | 0 (0%) | 0.019 |
| Tumor size(cm) | 2.95±0.73 | 2.09 ± 0.70 | < 0.001 |

Immunohistochemical staining was used to detect the expression of HIF-1α in normal pancreas specimens and in pancreatic cancer specimens from patients with or without diabetes (**Figure 1**). A higher HIF-1α expression was detected in the hyperglycemic group than in the normal pancreas specimens (**Figure 1A, B**). Representative immunohistochemical staining images of the expression of HIF-1α in specimens from pancreatic cancer patients are shown in **Figure 1C-E**. The HIF-1α expression levels in pancreatic cancer specimens from the hyperglycemic group and the euglycemic group are summarized in **Table 2**. There are 3 patients with negative HIF-1α in euglycemia group and 1 in hyperglycemia group.

**Table 2.** The expression level of HIF-1α in pancreatic cancer specimens.

| HIF-1α expression | | Hyperglycemia（18） | Euglycemia（28） | *P* |
| --- | --- | --- | --- | --- |
| -/+ | 5 | | 13 |  |
| ++ | 5 | | 12 |  |
| +++ | 8 | | 3 | <0.05 |

These results indicated that pancreatic cancer patients with diabetes were more likely to experience tumor progression. Hyperglycemia might promote HIF-1α protein synthesis and a hypoxic microenvironment.

***The effect of hyperglycemia on microenvironment hypoxia.***

Based on the clinical findings, we next conducted experiments to ascertain whether hyperglycemia promotes microenvironment hypoxia that then aggravates pancreatic cancer invasion in vivo. To induce diabetes in mice, 175 mg/kg STZ was injected into the peritoneal cavity of nude mice. The weight and blood glucose of the mice were monitored. As shown in **Table 3**, the STZ injection group showed higher levels of blood glucose from 2 to 8 weeks. After 8 weeks, the mice were sacrificed, and the pancreatic tissues were collected and embedded. HE staining showed morphology changes in diabetic mice, including swelling in pancreatic acinar cells and broadening in the lobule intervals, compared with those in the control group (**Figure 2A, B**). Transmission electron microscopy was used to identify the morphology of cell subsets **(****Figure 2C, D)**. Pancreatic acinar cells in diabetic mice showed an obvious expansion of the endoplasmic reticulum, increased nuclear gaps and chromatin close to the cellular membrane in some acinar cells. The immunohistochemistry staining showed that the HIF-1α expression level was higher in the hyperglycemic group than in the euglycemic group (**Figure 2E, F**). Our results indicated that a hyperglycemic environment might induce microenvironmental hypoxia.

**Table 3. Effect of hyperglycemia on nude mice weight and tumor growth, ascites and liver metastasis.**

| Mice | | Hyperglycemia（10） | Euglycemia（10） | *P* |
| --- | --- | --- | --- | --- |
| Blood Glucose (2w) ^*^  Blood Glucose (8w) ^*^  Weight (8w) ^*^  Tumor volume (mm^3^) ^*^ | 24.59 ± 2.30  22.58 ± 2.24  20.24 ± 1.85  746.50 ± 210.76 | | 5.52 ± 1.33  5.48 ± 1.47  25.72 ± 1.38  1824.70 ± 878.36 | <0.001  <0.001  <0.001  0.001 |
| Ascites | 6 | | 1 | 0.057 |
| Liver metastasis ^*^ | 5 | | 0 | 0.033 |

***Hyperglycemia promotes pancreatic cancer progression and tumor microenvironment hypoxia in nude mice.***

Our clinical data indicate that pancreatic cancers are more aggressive in patients who have hyperglycemia. To verify our clinical findings, an orthotopic xenograft model was used with or without intraperitoneal injection of STZ. The average tumor volume was greater in diabetic mice than in euglycemic mice (**Table 3, Figure 3A-D**). Diabetic mice were more likely to combine ascites (**Figure 3E-H**) with liver metastasis (**Figure 3I, J**) than mice in the control group.

Hypoxyprobe-1, a type of pimonidazole hydrochloride, was used as a specific marker for hypoxia [14]. The immunohistochemistry results showed that the expression areas for Hypoxyprobe-1 and HIF-1α (**Figure 4A**) in the diabetic, orthotopic xenograft group were larger than in the control group. Immunoblotting results revealed that the expression of HIF-1α and MMP-9 in the diabetic group was higher than in the control group (**Figure 4B**). These data suggested that a hyperglycemic environment could exacerbate hypoxia and promote tumor progression in the pancreatic orthotopic xenograft model.

***The effect of high glucose and CoCl_2_ on the expression of HIF-1α in pancreatic cancer cells.***

To explore the possible relationship between hyperglycemia and hypoxia, we examined the effects of high glucose concentrations and the expression of HIF-1α in pancreatic cancer cells. Our results showed that the expression levels of HIF-1α in BxPC-3 cancer cells increased in a dose-dependent manner in response to high glucose concentrations compared with normal physiological glucose levels (5.5 mM glucose) (**Figure 5A**). Cobalt chloride (CoCl_2_), a mimetic agent used to induce cellular responses mediated by hypoxia *in vitro*, stabilizes HIF-1α by inhibiting prolyl hydroxylase enzymes [15]. Both hypoxia and CoCl_2_ induce the transcriptional responses of HIF-1α to reduced oxygen tension [16]. Incubation with 0 to 150 μM CoCl_2_ induced the expression of HIF-1α in BxPC-3 cells in a dose-dependent manner. Although 200 μM CoCl_2_ also increased HIF-1α expression, the efficiency had declined (**Figure 5B**). Therefore, a treatment concentration of 150 μM CoCl_2_ was used in the subsequent experiments. As shown in **Figure 5C**, incubation for 6 to 72 h with 150 μM CoCl_2_ promoted the time-dependent expression of HIF-1α. Interestingly, our results indicated that a low glucose concentration augmented HIF-1α production in CoCl_2_-treated BxPC-3 cells compared with a high glucose concentration (**Figure 5D)**.

***HIF-1α is involved in high glucose-induced pancreatic cancer invasion and migration.***

To confirm that high glucose-induced HIF-1α influences the invasive and migratory abilities of pancreatic cancer cells, we used HIF-1α siRNA to knock down HIF-1α. Three siRNA sequences were designed and efficiency of these siRNAs was evaluated by Western blotting (**Figure 6A**). And HIF-1α siRNA^#1^ was chosen for further experiments for its high quality effect on knockdown HIF-1α expression. We found that the increased BxPC-3 cell invasion and migration in the presence of high glucose was significantly inhibited by the HIF-1α knockdown (**Figure 6B, C**). Additionally, high glucose induced-MMP9 production was down-regulated in the HIF-1α siRNA group compared with the control siRNA group (**Figure 6D**). These data suggest that a high glucose level contributes to increased pancreatic cancer invasion and migration in a HIF-1α-dependent manner.

**Discussion**

Pancreatic cancer is among the deadliest types of malignant digestive carcinoma. The 5-year relative survival rate is currently 8% because pancreatic cancer is commonly diagnosed in an advanced stage [17]. The total deaths due to pancreatic cancer are projected to surpass breast, prostate and colorectal cancers to become the second leading cause of cancer-related deaths by 2030 [18]. Identification of etiological factors could enable the early detection of this fatal disease so that it would be more amenable to treatment. Diabetes mellitus is among the small number of known potential risk factors for pancreatic cancer. A recent prospective study of 0.5 million Chinese adults showed that diabetes was associated with an almost two-fold increased risk of pancreatic cancer and that every 1 mmol/l increase in blood glucose level led to an increase of 15% in the incidence of pancreatic cancer [19].

Hyperglycemia and hyperinsulinemia are related factors that promote dysplasia and neoplasia in the pancreas [5]. Diabetes and the accompanying hyperglycemia lead to chronic inflammation and increased cancer risk [20]. Our previous studies showed that hyperglycemia could promote the progression of pancreatic cancer. Our studies demonstrated that high glucose *in vitro* might be regarded as an accelerator to increase cancer cell proliferation by enhancing the glial cell line-derived neurotrophic factor (GDNF)/RET or epidermal growth factor (EGF)/EGFR signaling pathways [21-22]. We also showed that high glucose could enhance the migratory and invasive abilities of pancreatic cancer cells through superoxide dismutase (SOD)-induced production of hydrogen peroxide (H_2_O_2_) via the activation of the ERK and p38 MAPK signaling pathways [23]. Our studies proved that hyperglycemic mice contained a higher plasma H_2_O_2_ level, which induced epithelial-mesenchymal transition (EMT) and promoted the metastatic activity of pancreatic orthotopic transplantation tumors, than euglycemic mice [7]. In addition, our results showed that a hyperglycemic tumor microenvironment induced perineural invasion (PNI) in pancreatic cancer through the impairment of nerve construction, formation of a low-resistance pathway of PNI by cancer cells and enhancement of the interaction between tumor cells and nerves [8].

A recent study has shown that hyperglycemia can induce cellular hypoxia and the production of mitochondrial reactive oxygen species, which in turn promote hyperglycemic damage in a coordinated manner [9]. Hypoxia exists in the microenvironment of solid tumors, including pancreatic cancer, and it plays an important role in tumor progression and metastasis [11]. In the current study, we observed that hyperglycemia could induce microenvironment hypoxia in pancreatic normal parenchyma and in tumor tissue. Hyperglycemia promotes the growth, invasion and metastasis of pancreatic cancer. The expression of hypoxia-inducible factor-1α (HIF-1α) in pancreatic tumors was higher in hyperglycemic conditions than in euglycemic conditions. The role of high glucose concentrations in promoting pancreatic cancer invasion involves the up-regulation of HIF-1α. Inhibition of HIF-1α by siRNA reversed the effect of high glucose on pancreatic cancer cells.

Hypoxia is not only a consequence of unrestrained fast tumor growth; it also plays a vital role in promoting tumor proliferation, invasion, EMT, distant metastasis and resistance to therapy [11-24]. The tumor stroma is a dynamic environment, which includes inflammatory cells, pancreatic stellate cells (PSC), endothelial cells, extracellular matrix (ECM), growth factors and cytokines. Hypoxia could affect stromal cells by promoting the activation of PSCs and the secretion of specific ECM components that induce the generation of the extensive desmoplastic stroma characteristic of pancreatic cancer [25]. HIF-1α is the most important transcription factor induced by intratumoral hypoxia, and it predicts poor outcomes for pancreatic cancer patients [26]. The expression of HIF-1α is also linked with pancreatic cancer progression, angiogenesis, EMT and metastasis [24]. HIF-1α is able to recruit monocytes and macrophages by promoting chemical chemokine 2 (CCL2) secretion, which accelerates the activation of PSCs and enhances tumor progression [27].

Hyperglycemic conditions and cellular hypoxia are intimately related. In tumor cells, high glucose levels promote HIF-1α expression under both normoxic and hypoxic conditions [12]. In the streptozotocin (STZ)-induced diabetic mouse model, glomerular mesangial cells showed a significant increase in HIF-1α expression in the nucleus. In cultured mesangial cells, high glucose levels could also enhance the expression of HIF-1α [28]. Yan et al. showed that high glucose (30 mM) up-regulated the protein level of HIF-1α, increased the transcriptional activity of HIF-1α and promoted the expression of vascular endothelial growth factor (VEGF), a downstream gene of HIF-1 in the endothelial cells [29]. Our results showed that hyperglycemia could induce hypoxia and increase the expression of HIF-1α in the normal pancreas and in pancreatic cancer.

**Conclusion**

In summary, our results indicate that pancreatic cancer, specifically the tumor within the pancreatic parenchyma, is more aggressive in a microenvironment combining hypoxia with hyperglycemia. High glucose levels can up-regulate HIF-1α expression, which promotes metastasis of pancreatic cancer. Our findings may provide new insight into the relationship between diabetes mellitus and pancreatic cancer. Managing hyperglycemia-induced HIF-1α might be a novel strategy for the treatment of pancreatic cancer. Our findings warrant further investigation of this possibility.

**Abbreviations**

EMT: epithelial-mesenchymal transition; HIF-1α: hypoxia-inducible factor 1α; STZ: streptozotocin; TEM: transmission electron microscopy; CoCl2: cobalt chloride; GDNF: glial cell line-derived neurotrophic factor; EGF: epidermal growth factor; SOD: superoxide dismutase; H2O2: hydrogen peroxide; PNI: perineural invasion; PSC: pancreatic stellate cells; ECM: endothelial cells, extracellular matrix; CCL2: chemical chemokine 2; VEGF: vascular endothelial growth factor

**Ethics approval and consent to participate**

The study protocol and consent forms conform to the Declaration of Helsinki and were approved by the Ethical Review Board (ERB) Committee of The First Affiliated Hospital of Xi’an Jiaotong University, China. Written informed consent was obtained from all the participants. Animal experimental protocols were approved by the Ethical Committee of the First Afﬁliated Hospital of Xi’an Jiaotong University, Xi’an, China.

**Conflict of interest**

All authors have declared no conflicts of interest.

**Funding**

This work was supported by grants from the National Natural Sciences Foundation of China (81472248, 81672434 and 81702916).

**Authors’ contributions**

WL, HL, QM, JM designed the study; WL, HL, WQ, LC, BY, LH, QX, QM, JM carried out experiments. WL, HL, JM analyzed the data. WL, JM wrote the manuscript. WL, QM, JM supervised and revised the manuscript. All authors had final approval of the submitted and published versions.

**References**

1. Siegel RL, Miller KD, Jemal A. Cancer statistics, 2018. CA Cancer J Clin. 2018; 68: 7-30.
2. He Y, Zheng R, Li D, Zeng H, Zhang S, Chen W. Pancreatic cancer incidence and mortality patterns in China, 2011. Chin J Cancer Res. 2015; 27: 29-37.
3. Torgeson A, Garrido-Laguna I, Tao R, Cannon GM, Scaife CL, Lloyd S. Value of surgical resection and timing of therapy in patients with pancreatic cancer at high risk for positive margins. ESMO Open. 2018; 3: e000282.
4. Pourhoseingholi MA, Ashtari S, Hajizadeh N, Fazeli Z, Zali MR. Systematic review of pancreatic cancer epidemiology in Asia-Pacific Region: major patterns in GLOBACON 2012. Gastroenterol Hepatol Bed Bench. 2017; 10: 245-257.
5. Eibl G, Cruz-Monserrate Z, Korc M, Petrov MS, Goodarzi MO, Fisher WE, Habtezion A, Lugea A, Pandol SJ, Hart PA, Andersen DK. Diabetes Mellitus and Obesity as Risk Factors for Pancreatic Cancer. J Acad Nutr Diet. 2017; doi: 10.1016/j.jand.2017.07.005.
6. Tan J, You Y, Guo F, Xu J, Dai H, Bie P. Association of elevated risk of pancreatic cancer in diabetic patients: A systematic review and meta-analysis. Oncol Lett. 2017; 13: 1247-1255.
7. Li W, Zhang L, Chen X, Jiang Z, Zong L, Ma Q. Hyperglycemia Promotes the Epithelial-Mesenchymal Transition of Pancreatic Cancer via Hydrogen Peroxide. Oxid Med Cell Longev. 2016; 2016: 5190314.
8. Li J, Ma J, Han L, Xu Q, Lei J, Duan W, Li W, Wang F, Wu E, Ma Q, Huo X. Hyperglycemic tumor microenvironment induces perineural invasion in pancreatic cancer. Cancer Biol Ther. 2015; 16: 912-921.
9. Sada K, Nishikawa T, Kukidome D, Yoshinaga T, Kajihara N, Sonoda K, Senokuchi T, Motoshima H, Matsumura T, Araki E. Hyperglycemia Induces Cellular Hypoxia through Production of Mitochondrial ROS Followed by Suppression of Aquaporin-1. PLoS One. 2016; 11: e0158619.
10. Tiffon C. Histone Deacetylase Inhibition Restores Expression of Hypoxia-Inducible Protein NDRG1 in Pancreatic Cancer. Pancreas. 2018; 47: 200-207.
11. Wei H, Xu Z, Liu F, Wang F, Wang X, Sun X, Li J. Hypoxia induces oncogene yes-associated protein 1 nuclear translocation to promote pancreatic ductal adenocarcinoma invasion via epithelial-mesenchymal transition. Tumour Biol. 2017; 39: 1010428317691684.
12. Dehne N, Hintereder G, Brune B. High glucose concentrations attenuate hypoxia-inducible factor-1 alpha expression and signaling in non-tumor cells. Exp Cell Res. 2010; 316: 1179-1189.
13. Livak KJ, Schmittgen TD. Analysis of relative gene expression data using real-time quantitative PCR and the 2(-Delta Delta C(T)) Method. Methods. 2001; 25: 402-408.
14. Yu J, Liang F, Huang H, Pirttiniemi P, Yu D. Effects of loading on chondrocyte hypoxia, HIF-1alpha and VEGF in the mandibular condylar cartilage of young rats. Orthod Craniofac Res. 2018; 21: 41-47.
15. Ho VT, Bunn HF. Effects of transition metals on the expression of the erythropoietin gene: Further evidence that the oxygen sensor is a heme protein. Biochem Bioph Res Co. 1996; 223: 175-180.
16. Li S, Zhang J, Yang H, Wu CH, Dang XT, Liu YY. Copper depletion inhibits CoCl2-induced aggressive phenotype of MCF-7 cells via downregulation of HIF-1 and inhibition of Snail/Twist-mediated epithelial-mesenchymal transition. Sci Rep. 2015; 5: 12410.
17. Siegel RL, Miller KD, Jemal A. Cancer Statistics, 2017. CA Cancer J Clin. 2017; 67: 7-30.
18. Rahib L, Smith BD, Aizenberg R, Rosenzweig AB, Fleshman JM, Matrisian LM. Projecting cancer incidence and deaths to 2030: the unexpected burden of thyroid, liver, and pancreas cancers in the United States. Cancer Res. 2014; 74: 2913-2921.
19. Pang Y, Kartsonaki C, Guo Y, Bragg F, Yang L, Bian Z, Chen Y, Iona A, Millwood IY, Lv J, Yu C, Chen J, Li L, Holmes MV, Chen Z. Diabetes, plasma glucose and incidence of pancreatic cancer: A prospective study of 0.5 million Chinese adults and a meta-analysis of 22 cohort studies. Int J Cancer. 2017; 140: 1781-1788.
20. Chang SC, Yang WV. Hyperglycemia, tumorigenesis, and chronic inflammation. Crit Rev Oncol Hematol. 2016; 108: 146-153.
21. Han L, Ma Q, Li J, Liu H, Li W, Ma G, Xu Q, Zhou S, Wu E. High glucose promotes pancreatic cancer cell proliferation via the induction of EGF expression and transactivation of EGFR. PLoS One. 2011; 6: e27074.
22. Liu H, Ma Q, Li J. High glucose promotes cell proliferation and enhances GDNF and RET expression in pancreatic cancer cells. Mol Cell Biochem. 2011; 347: 95-101.
23. Li W, Ma Z, Ma J, Li X, Xu Q, Duan W, Chen X, Lv Y, Zhou S, Wu E, Ma Q, Huo X. Hydrogen peroxide mediates hyperglycemia-induced invasive activity via ERK and p38 MAPK in human pancreatic cancer. Oncotarget. 2015; 6: 31119-31133.
24. Chen S, Chen JZ, Zhang JQ, Chen HX, Yan ML, Huang L, Tian YF, Chen YL, Wang YD. Hypoxia induces TWIST-activated epithelial-mesenchymal transition and proliferation of pancreatic cancer cells in vitro and in nude mice. Cancer Lett. 2016; 383: 73-84.
25. Yuen A, Diaz B. The impact of hypoxia in pancreatic cancer invasion and metastasis. Hypoxia (Auckl). 2014; 2: 91-106.
26. Ye LY, Zhang Q, Bai XL, Pankaj P, Hu QD, Liang TB. Hypoxia-inducible factor 1alpha expression and its clinical significance in pancreatic cancer: a meta-analysis. Pancreatology. 2014; 14: 391-397.
27. Li N, Li Y, Li Z, Huang C, Yang Y, Lang M, Cao J, Jiang W, Xu Y, Dong J, Ren H. Hypoxia Inducible Factor 1 (HIF-1) Recruits Macrophage to Activate Pancreatic Stellate Cells in Pancreatic Ductal Adenocarcinoma. Int J Mol Sci. 2016; 17: 1-12
28. Isoe T, Makino Y, Mizumoto K, Sakagami H, Fujita Y, Honjo J, Takiyama Y, Itoh H, Haneda M. High glucose activates HIF-1-mediated signal transduction in glomerular mesangial cells through a carbohydrate response element binding protein. Kidney Int. 2010; 78: 48-59.
29. Yan JQ, Zhang ZY, Shi HL. HIF-1 is involved in high glucose-induced paracellular permeability of brain endothelial cells. Cell Mol Life Sci. 2012; 69: 115-128.

**Figure Legends**

Figure 1. Representative images showing HIF-1α expression in normal pancreas specimens and in pancreatic cancer specimens. Immunohistochemical staining of normal pancreas specimens for HIF-1α. A, the euglycemic group and B, the hyperglycemic group; C, D, and E show different expression levels of HIF-1α in pancreatic cancer specimens. C, a low expression level of HIF-1α (+), D, an intermediate expression level of HIF-1α (++) and E, a high expression level of HIF-1α (+++). (HIF-1α antibody concentration 1:200; 200× magnification).

Figure 2. The effect of hyperglycemia on the normal pancreas. To induce hyperglycemia, 175 mg/kg STZ was injected into the peritoneal cavity of each mouse. Mice were sacrificed after 8 weeks. The H&E staining and transmission electron microscopy showed the morphology changes in the pancreas (A, C, the euglycemic group and B, D, the hyperglycemic group). Immunohistochemical staining of normal pancreas specimens for HIF-1α: E, the euglycemic group and F, the hyperglycemic group.

Figure 3. The effects of hyperglycemia on tumor growth, ascites generation and liver metastasis in nude mice.

Macroscopic appearance of solid tumors after the mice were sacrificed: A, B, euglycemic group and C, D, the hyperglycemic group. Ascites generation and liver metastasis in nude mice: E, F, I, the euglycemic group and G, H, J, the hyperglycemic group.

Figure 4. Effects of hyperglycemia on microenvironment hypoxia in pancreatic cancer. A, Immunohistochemistry was performed to compare the expression of Hypoxyprobe-1 and HIF-1α in the euglycemic group and hyperglycemic group of orthotopic nude mice. B, The protein levels of HIF-1α and MMP-9 in pancreatic tumor tissues with different serum glucose levels were analyzed using Western blotting. **P* < 0.05 compared to the euglycemic group.

Figure 5. Effects of high glucose and CoCl_2_ on HIF-1α expression in BxPC-3 cells. A, The effect of glucose on the protein level of HIF-1α. BxPC-3 cells were treated with different concentrations of glucose for 72 h, and the protein level of HIF-1α was evaluated. B, The effect of CoCl_2_ on the protein levels of HIF-1α. C, The effect of 150 μM CoCl_2_ on the protein level of HIF-1α at the indicated times. D, The effect of CoCl_2_ on HIF-1α expression in both normal glucose and high glucose conditions. **P* < 0.05 compared to the control group.

Figure 6 HIF-1α siRNA abolished the effects of high glucose-mediated invasion and migration of pancreatic cancer cells. A, Western blotting were used to evaluated the efficiency of siRNAs targeting HIF-1α in BxPC-3cells. B, The effect of HIF-1α knockdown on BxPC-3 cell invasion. The images show the bottom side of the filter inserts with stained cells that migrated through the filter pores at 48 h. The number of migrated cells was quantified by counting the cells from 10 random fields at ×200 magnification. C, The effect on cancer cell migration in response to HIF-1α knockdown. The conﬂuent monolayer was wounded with a sterile pipette tip, and the cells were allowed to migrate for 24 h. D, qRT-PCR and Western blotting were used to test the effect of the HIF-1α knockdown on MMP-9 expression at both mRNA and protein levels.
